# Proteomic analysis of *Daphnia magna* hints at molecular pathways involved in defensive plastic responses

**DOI:** 10.1186/1471-2164-15-306

**Published:** 2014-04-24

**Authors:** Kathrin A Otte, Thomas Fröhlich, Georg J Arnold, Christian Laforsch

**Affiliations:** 1 Department Biology II,, Ludwig-Maximilians-University Munich, Großhaderner Str. 2, 82152 Planegg-Martinsried, Germany; 2 Laboratory for Functional Genome Analysis (LAFUGA), Gene Center, Ludwig-Maximilians-University Munich, Feodor-Lynen-Str. 25, 81377 München, Germany; 3 Animal Ecology I, Bayreuth University, 95440 Bayreuth, Germany

**Keywords:** *Daphnia*, Phenotypic plasticity, Inducible defence, Predator-prey interaction, 2D-DIGE, Proteomics

## Abstract

**Background:**

Phenotypic plasticity in defensive traits occurs in many species when facing heterogeneous predator regimes. The waterflea *Daphnia* is well-known for showing a variety of these so called inducible defences. However, molecular mechanisms underlying this plasticity are poorly understood so far. We performed proteomic analysis on *Daphnia magna* exposed to chemical cues of the predator *Triops cancriformis*. *D. magna* develops an array of morphological changes in the presence of *Triops* including changes of carapace morphology and cuticle hardening.

**Results:**

Using the 2D-DIGE technique, 1500 protein spots could be matched and quantified. We discovered 179 protein spots with altered intensity when comparing *Triops* exposed animals to a control group, and 69 spots were identified using nano-LC MS/MS. Kairomone exposure increased the intensity of spots containing muscle proteins, cuticle proteins and chitin-modifying enzymes as well as enzymes of carbohydrate and energy metabolism. The yolk precursor protein vitellogenin decreased in abundance in 41 of 43 spots.

**Conclusion:**

Identified proteins may be either directly involved in carapace stability or reflect changes in energy demand and allocation costs in animals exposed to predator kairomones. Our results present promising candidate proteins involved in the expression of inducible defences in *Daphnia* and enable further in depth analysis of this phenomenon.

## Background

Phenotypic plasticity describes the ability of a genotype to express different phenotypes in response to varying environmental conditions [1,2]. Given that phenotypic plasticity is an important adaptation to face heterogeneous environments it is a fundamental aspect of the ecology and evolution of a broad range of organisms [3].

One frequently changing biotic condition, which strongly influences organisms’ fitness and abundance in an ecological community context, is predation [4]. Phenotypic plasticity in defensive traits, so called inducible defences, occur in many species throughout invertebrate, vertebrate and plant taxa [5]. They are especially common in aquatic environments, where prey species can easily detect chemical cues (kairomones) released by predators [6].

Important key stone species of fresh water environments are waterfleas (*Daphnia*: Crustacea). The biology of these animals was studied over the past 250 years [7], resulting in a large amount of literature documenting their ecological diversity. With the help of the *Daphnia Genomics Consortium* (https://wiki.cgb.indiana.edu/display/DGC/Home), *Daphnia* is now one of the leading model organisms in evolutionary and ecological functional genomics. With the published genome sequence of *Daphnia pulex*[8] and the available pre-release of the *Daphnia magna* genome sequence (https://wiki.cgb.indiana.edu/display/DGC/Daphnia+magna+Genome), the American National Institutes of Health (NIH) has added *Daphnia* to their list of model organisms for biomedical research (http://www.nih.gov/science/models/daphnia/).

*Daphnia* shows a multitude of inducible defences in response to changing predator regimes and hence serves as textbook example for phenotypic plasticity in defensive traits (reviewed in [9]). These defences include life history shifts like altered size or age at maturity [10-12], modifications of behaviour, e.g. diel vertical migration [13-15] and morphological changes including the formation of spine-like structures and helmets [16-18]. Also so called hidden morphological defences, which increase the stability of the carapace, were found [19-21].

The description of the *D. pulex* genome unravelled large arrays of environmental specific genes [8], which may be the key players in the formation of phenotypic plastic traits [22]. These genes often reside within the elevated number of tandem duplications, a striking feature of the *D. pulex* genome [8]. The same seems to be true for the genome of *D. magna* (Colbourne, pers. commun.). However, as molecular tools and genomic resources for *Daphnia* have only recently become available, the analysis of molecular mechanisms underlying inducible defences in *Daphnia* exposed to predator kairomones is still in its infancy (summarised in [23]). Up to date, only few studies have been conducted using either candidate gene/protein approaches [24-26] or a microarray approach based on stress and life stage specific cDNA libraries [27] in *D. magna*.

In these studies, genes involved in protein biosynthesis, protein catabolism and protein folding [26,27] showed different RNA expression patterns between *D. magna* defended against fish or *Chaoborus* and a control group. Also heat shock proteins, confirmed by western blot analysis, were found to be involved in the anti-predator defence of *D. magna*, being more abundant after short-term exposure [25] but less abundant after long-term exposure to fish kairomones [24]. Furthermore, two proteins of the cytoskeleton, actin and alpha tubulin, were affected [24].

The availability of enhanced genomic resources for *Daphnia* not only facilitates candidate gene approaches but also enables holistic approaches. In contrast to candidate approaches, holistic experiments may elucidate unpredicted key players involved in trait formation and regulation of inducible defences in *Daphnia*. Holistic proteomic analysis is especially suitable, as proteins are the typical effectors of biological functions and protein abundance is not necessarily well correlated with the corresponding mRNA level (e.g. [28,29]).

**Figure 1:**
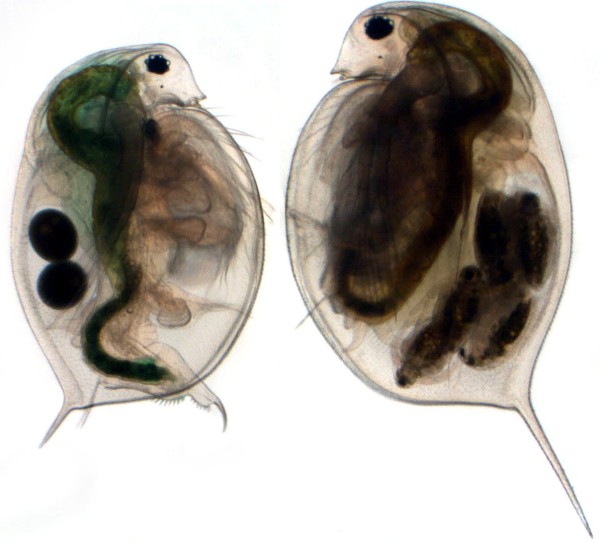
**Inducible defence in *D. magna*.** Adult *D. magna* showing increased bulkiness after *Triops* induction (right) compared to control animal (left). (Photo: M. Rabus).

In the present study, we used the predator-prey system of *Triops cancriformis* and *Daphnia magna* for analysis of proteins involved in the formation of inducible defences. *D. magna* is a common species found in temporary and permanent ponds spreading from temperate regions to arid areas in the Holarctic and Africa [30]. This species shows inducible morphological defences in response to kairomones released by *T. cancriformis*. These morphological changes result in an increased bulkiness (increased body length, increased body width, increased tail spine length; see Figure 1) and are known to serve as an effective defence against *Triops* predation [31,32]. In addition, *D. magna* develops hidden morphological defences when exposed to *Triops* kairomones, which consist of a harder and thicker cuticle and an increased diameter of cuticle pillars, and therefore enhance carapace stability [21].

## Results

We have studied differentially abundant proteins in *D. magna* exposed to kairomones of the predator *T. cancriformis*, which is known to induce phenotypic plastic defensive structures in this species [31], and a control group not exposed to predator kairomones. Performing proteomic analysis of adult *Daphnia* is a challenging task due to very strong proteolytic activity [33-36], which most likely results from proteases expressed in the digestive tract [37]. To avoid proteolytic degradation of protein lysates, we sampled late stage *D. magna* embryos featuring reduced protease activity. The sensitive period in *Daphnia* for perceiving chemical cues released by predators and for the formation of defensive traits is known to happen during embryonic development [38]. Preliminary experiments proved the same for *D. magna* exposed to *Triops* rendering late embryonic stages perfectly suitable for proteomic analysis.

Proteomic 2D-DIGE analysis and mass spectrometric analysis of abundance altered spots resulted in identification of 69 protein spots with 23 being more intense in kairomone exposed animals and 46 less intense. Mass spectrometric data, summarised spot data and further details are provided in the supplementary files (see Additional files 1, 2 and 3).

**Figure 2:**
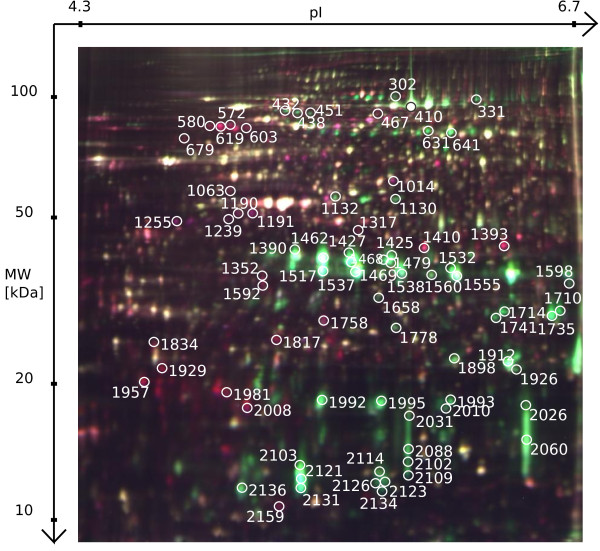
**2D DIGE gel for comparing *Triops *exposed and control *D. magna *embryos.** Spots with more abundant proteins in the kairomone exposed group are displayed in red (Cy5 labelled), spots with more abundant proteins in the control group are displayed in green (Cy3 labelled). Spots marked with Spot ID showed significantly different intensity and were successfully identified. Spot IDs not listed in Table 1 or Table 2 refer to vitellogenin-related spots.

**Figure 3:**
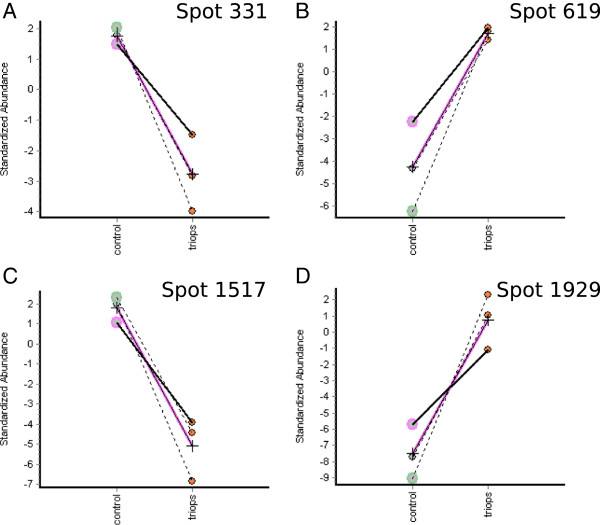
**Examples for normalised DIGE intensity ratios.** Normalisation was done according to internal pooled standard (IPS), here an abundance of e.g. 2 indicates that abundance is 2 of IPS abundance whereas -2 means 1/2 of IPS abundance. They serve as indicators for changes in protein abundance in kairomone exposed *D. magna* and in the control group for: Spot 331 – STAT Protein **(A)**; Spot 619 – Chitin deacetylase 2A **(B)**; Spot 1517 – Vitellogenin **(C)** and Spot 1929 – Cuticle Protein **(D)**.

**Figure 4:**
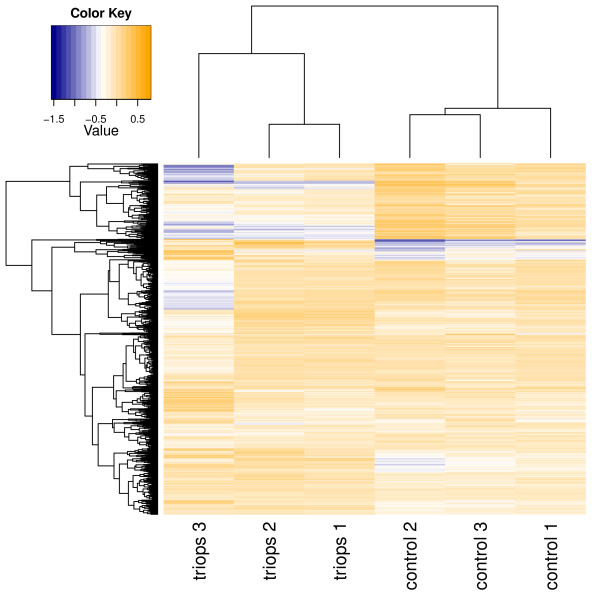
**Hierarchical clustering heat map of all protein spots present in at least two biological replicates.** Graph was created with R using the heatmap.2 function of package gplots. Rows indicate proteins whereas columns represent biological replicates of *Triops* kairomone exposed animals (*triops*) and control group (*control*).

In detail, three biological replicates of *Triops* kairomone exposed animals and three biological replicates of a control group were compared using three 2D-DIGE gels. The gel images were of high-quality (see Figure 2 and also Figure 3) with all three gels showing highly reproducible spot patterns (see Additional file 4). In an unsupervised hierarchical cluster analysis, spot patterns clustered in two distinct groups, each containing solely gels from *Triops* kairomone exposed animals and controls, respectively (see Figure 4).

**Table 1:** **More intense spots for kairomone exposed *Daphnia *** **in 2D DIGE analysis (n=3)**

**Spot**	**GeneID**	**UniprotID**	**Protein Name (Organism)**	**FlybaseID**	**Ratio**	**Mw**	**Mw**	**pI**	**pI**
						**theo**	**exp**	**theo**	**exp**
1191	daphmag3mtv3l7094t1	Q9NA03	Actin (Daphnia magna)	FBgn0000046	14.3 ± 1.8	42	51	5.3	5
2008	daphmag3mtv3l18463t2	E9FZ29	Putative uncharacterized protein (Daphnia pulex) Nucleoside diphosphate kinase (Orseolia oryzae)	FBgn0000150	14 ± 0.2	17	18	6.2	5
1255	daphmag3mtv3l7094t1	Q9NA03	Actin (Daphnia magna)	FBgn0000046	13.2 ± 1.6	42	48	5.3	4.6
1929	daphmag3mtv3l7285t1	E9GDV0	Putative uncharacterized protein (Daphnia pulex) Cuticle protein (Artemia franciscana)	FBgn0033869	10.2 ± 0.7	19	22	5.7	4.5
1981	daphmag3mtv3l8582t2	E9HPK7	Putative uncharacterized protein (Daphnia pulex) Cuticle protein1c (Daphnia magna)	FBgn0086900	9.2 ± 0.6	39	19	5.1	4.9
1817	daphmag3mtv3l7094t1	Q9NA03	Actin (Daphnia magna)	FBgn0000046	8.6 ± 0.7	42	26	5.3	5.2
572	daphmag3mtv3l9358t1	E9HBN5	Putative uncharacterized protein (Daphnia pulex) Chitin deacetylase 2A (Tribolium castaneum)	FBgn0261341	7.7 ± 0.1	59	81	5.2	4.9
572	daphmag3mtv3l7734t1	E9HBN3	Putative uncharacterized protein (Daphnia pulex) Chitin deacetylase 1 (Tribolium castaneum)	FBgn0260653	7.7 ± 0.1	62	81	5	4.9
619	daphmag3mtv3l9358t1	E9HBN5	Putative uncharacterized protein (Daphnia pulex) Chitin deacetylase 2A (Tribolium castaneum)	FBgn0261341	6.2 ± 0.1	59	79	5.2	5
1957	daphmag3mtv3l20379t3	E9HPJ8	Putative uncharacterized protein (Daphnia pulex) Cuticle protein1b (Daphnia magna)	FBgn0000551	5.3 ± 0.6	22	21	5.5	4.4
2159	daphmag3mtv3l10909t1	E9FQP0	ATP synthase subunit beta (Daphnia pulex)	FBgn0010217	5.2 ± 0.2	56	11	5.4	5.2
603	daphmag3mtv3l9358t1	E9HBN5	Putative uncharacterized protein (Daphnia pulex) Chitin deacetylase 2A (Tribolium castaneum)	FBgn0261341	4.6 ± 0.2	59	80	5.2	5
603	daphmag3mtv3l7734t1	E9HBN3	Putative uncharacterized protein (Daphnia pulex) Chitin deacetylase 1 (Tribolium castaneum)	FBgn0260653	4.6 ± 0.2	62	80	5	5
1393	daphmag3mtv3l21933t1	E9GF36	Glyceraldehyde-3-phosphate dehydrogenase (Daphnia pulex)	FBgn0001092	4.2 ± 0.3	19	43	5.9	6.4
1758	daphmag3mtv3l21417t1	E9HCF1	Putative uncharacterized protein (Daphnia pulex) Probable phosphomannomutase (Drosophila melanogaster)	FBgn0036300	4.2 ± 1	16	28	7.9	5.4
1063	daphmag3mtv3l10909t1	E9FQP0	ATP synthase subunit beta (Daphnia pulex)	FBgn0010217	3.9 ± 0	56	58	5.4	4.9
1239	daphmag3mtv3l7094t1	Q9NA03	Actin (Daphnia magna)	FBgn0000046	3.8 ± 1.1	42	49	5.3	4.9
467	daphmag3mtv3l4480t1	E9HSV9	Paramyosin (Daphnia pulex)	FBgn0003149	3.5 ± 0.1	104	85	5.5	5.7
679	daphmag3mtv3l9455t1	E9HEE5	Putative uncharacterized protein (Daphnia pulex) Troponin H isoform 1 (Apis mellifera)	FBgn0004028	3.5 ± 0.3	44	74	4.8	4.7
1410	daphmag3mtv3l8855t1	E9GJ13	Fructose-bisphosphate aldolase (Daphnia pulex)	FBgn0000064	3.5 ± 0.5	40	42	6.7	6
1834	daphmag3mtv3l7635t2	E9H1W5	Putative uncharacterized protein (Daphnia pulex) 14-3-3 zeta (Artemia franciscana)	FBgn0004907	3.5 ± 0	39	25	8.5	4.5
1190	daphmag3mtv3l7094t1	Q9NA03	Actin (Daphnia magna)	FBgn0000046	3.3 ± 0.7	42	51	5.3	5
1352	daphmag3mtv3l16198t1	E9GE24	Putative uncharacterized protein (Daphnia pulex) Retinol dehydratase (Danaus plexippus)	FBgn0033887	3 ± 0.1	39	45	6.3	6.5

Spots were identified with LC-MS/MS and annotated using blastp algorithm against NCBI nr database. Spots related to vitellogenin were not shown.

**Table 2:** **Less intense spots for kairomone exposed *Daphnia *** **in 2D DIGE analysis (n=3)**

**Spot**	**GeneID**	**UniprotID**	**Protein Name (Organism)**	**FlybaseID**	**Ratio**	**Mw**	**Mw**	**pI**	**pI**
						**theo**	**exp**	**theo**	**exp**
1658	daphmag3mtv3l7424t1	E9GTZ4	Putative uncharacterized protein (Daphnia pulex) Prohibitin protein WPH (Danaus plexippus)	FBgn0002031	-3.5 ± 0.2	30	32	5.8	5.7
331	daphmag3mtv3l10027t1	E9G1W0	Putative uncharacterized protein (Daphnia pulex) Signal transducer and activator of transcription (Artemia franciscana)	FBgn0016917	-4.1 ± 0.1	63	92	7.3	6.5
631	daphmag3mtv3l2732t1	E9GIU3	Putative uncharacterized protein (Daphnia pulex) Heat shock protein (Culex quinquefasciatus)	FBgn0026761	-10.1 ± 1.2	78	78	6.5	6

Spots were identified with LC-MS/MS and annotated using blastp algorithm against NCBI nr database. Spots related to vitellogenin were not shown.

By software assisted image analysis of 2D-DIGE gels, 1505 spots could be matched, i.e., corresponding spots of the three replicates were assigned in a supervised manner, and the intensity of all matched spots was quantified. 179 spots were found with different intensities between *Triops* exposed and control *Daphnia* (*p*≤0.05, *ratio*≥|3|). Out of these spots, 58 showed increased intensity in gels from *Triops* exposed animals whereas 121 showed decreased intensity.

87 spots were successfully identified using nano-LC MS/MS. Unambiguous identification of one single protein per spot was possible for 56 spots, while the majority of remaining spots contained contaminating fragments of the yolk protein precursor vitellogenin. The latter spots composed of peptides referring to more than one protein were only included in the bioinformatic analyses, if the total number of assigned peptides for one protein was at least three times higher than the number of all other assigned peptides. The corresponding protein was then regarded to represent the major component.

With respect to these classifications, we identified 69 protein spots in total. Out of this, 23 spots were more abundant in *Triops* exposed *D. magna* with 21 spots not containing vitellogenin (see Table 1). Of the remaining 46 spots, which were less abundant in *Triops* exposed *D. magna*, only 3 spots contained other proteins than vitellogenin (see Table 2). For vitellogenin-related spots, see the Additional file 2.

More abundant proteins of animals exposed to *Triops* kairomones (see Table 1) include proteins related to the cuticle (e.g. chitin deacetylase, different cuticle proteins), proteins involved in carbohydrate metabolism (glyceraldehyde-3-phosphate dehydrogenase, fructose-bisphosphate aldolase, ATP synthase), proteins related to the muscular system (paramyosin, troponin and actin), phosphorylation (nucleoside diphosphate kinase), glycosylation (phosphomannomutase) and a regulatory 14-3-3 *ζ* protein (see Table 1).

Less abundant proteins of animals exposed to *Triops* kairomones (see Table 2) include a protein responsible for larval development called Prohibitin, a transcription activator (STAT) and a heat shock protein (HSP70).

**Figure 5:**
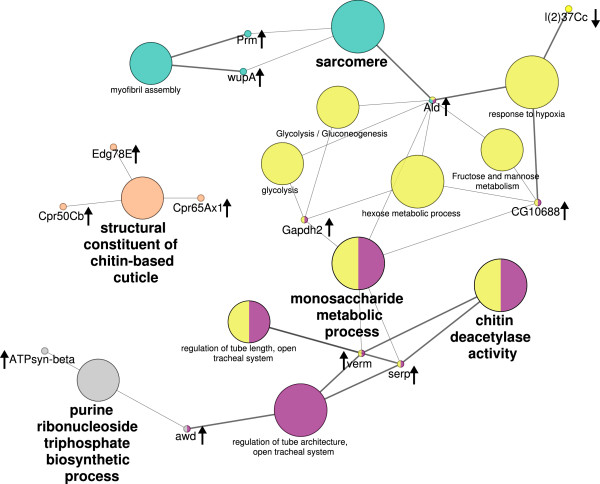
**Annotation term network created with ClueGo using functional annotation analysis (two-sided hypergeometric test, Benjamini-Hochberg-correction, kappa-score ≥0*.*3).** FlybaseIDs of proteins with increased and decreased abundance were searched against GO and KEGG databases. Small circles show involved genes and large circles refer to GO terms. Arrows next to gene names indicate decreased or increased abundance. Colours represent grouping of GO terms whereas size of circle and circle label illustrate the corrected p-value. Abbreviated Drosophila gene names correspond to the following protein names (compare also Tables 1 and 2): Ald – Fructose-Bisphosphate aldolase, ATPsyn-beta – ATP synthase beta, awd – Nucleoside diphosphate kinase, CG10688 – Phosphomannomutase, Cpr50Cb – Cuticle protein, Cpr65Ax1 – Cuticle protein 1c, Edg78E – Cuticle protein 1b, Gapdh2 – GAPDH, l(2)37Cc – Prohibitin, Prm – Paramyosin, serp – Chitin deacetylase 1, verm – Chitin deacetylase 2A, wupA – Troponin.

To find grouped protein annotation terms and to visualise their relationships, ClueGO network analysis [39] was conducted using the Gene Ontology and KEGG databases of *D. melanogaster* (see Figure 5). Four functional groups could be separated, which were related to either glycolysis, actin cytoskeleton, chitin deacetylase activity or nucleoside triphosphate biosynthetic processes.

Comparison of protein data to known tandem duplicated genes in *D. pulex* with three or more duplications resulted in matching of three proteins. One cuticle protein (FBgn0033869, 33 duplications), Actin (3 duplications) and vitellogenin (4 duplications) were found to be tandem duplicated in the *D. pulex* genome.

## Discussion

Predation is a key factor driving natural selection and therefore important for evolution of prey species and dynamics of prey communities [40]. As predator quantity and quality usually show heterogeneous patterns [41], prey species develop a variety of plastic defences in response to changing predator regimes [4]. Predator characteristics, e.g. prey-preference, feeding mechanism, predation strategy and habitat use, play an important role in shaping these plastic defences [42].

Particularly, *Daphnia* has to cope with a variety of size-selective predators [43]. Vertebrate predators like visually hunting fish are usually limited in the process of detecting the prey, whereas invertebrate predator like *Chaoborus* or *Triops* are often limited at the capturing, handling or ingestion step. Therefore, *Daphnia* coexisting with fish usually are smaller and more transparent [7] and show avoidance behaviour such as diel vertical migration [13-15]. In response to invertebrate predators, *Daphnia* often develops morphological defences (e.g. [16-18,31]), which impede capturing, handling or ingestion by the predator.

We studied the defensive responses of *D. magna* exposed to *T. cancriformis*, which consist of morphological changes resulting in an increased bulkiness (increased body length, increased body width, increased tail spine length; see Figure 1).

Proteins more abundant in kairomone exposed *Daphnia* were similar to proteins connected to regulation, carbohydrate metabolism, biosynthetic processes, muscular system or the cuticle (see Table 1). The majority of less abundant proteins was identified as different isoformes of the yolk protein precursor vitellogenin. Three proteins of this data-set (cuticle protein, actin, vitellogenin) are known to be tandem-duplicated in the genome of *D. pulex*. Tandem-duplicated genes are thought to play an important role in the formation of phenotypic plastic traits [22].

### Proteins involved in the formation of inducible defences regulate cell proliferation, participate in signalling pathways and facilitate protein folding

Two proteins with regulatory function, 14-3-3 *ζ* and phosphomannomutase, were of higher abundance in *D. magna* embryos exposed to *Triops* kairomones in our study. 14-3-3 proteins belong to a family of proteins well conserved among eukaryotes. Two of these isoformes, *ε* and *ζ*, have also been identified in *D. melanogaster*[44] and the silkworm *Bombyx mori*[45] and were expressed throughout a variety of life stages and in various tissues. 14-3-3 *ζ* binds to a large number of partners by recognition of a phosphoserine or phosphothreonine motif and is known to modulate their activity. Phosphomannomutase is an enzyme converting mannose-1-phosphate to mannose-6-phosphate and vice versa. It is therefore important for GDP-mannose synthesis, a molecule involved in glycosylation of proteins. The most similar protein in Drosophila, CG10688, is known to be involved in hypoxia-induced inhibition of protein translation [46]. In kairomone exposed *D. magna*, phosphomannomutase may therefore provide substrates important for signalling pathways involved in the formation of inducible defences.

Additionally, three proteins with regulatory characteristics, heat shock protein 78 kDa, prohibitin and a transcription activator (STAT), were less abundant in *D. magna* embryos exposed to *Triops* kairomones.

Heat shock proteins (HSP) act as chaperones facilitating protein folding and unfolding and play an important role in both, normal cellular homoeostasis and stress response [47]. Pijanowska and Kloc [24] found a decrease in the levels of HSP40, HSP60 and HSP70 when exposing *D. magna* from birth until first reproduction to either fish or *Chaoborus* kairomones. These findings corresponds to our findings, we also found a strong decrease in a 78 kDa HSP in *Daphnia* long-term exposed to *Triops* kairomones. Reducing HSP expression may save resources under long-term stressful conditions [48]. In addition, another study using *D. magna* shows that animals with a high tolerance against cadmium exposure display lower levels of HSP70 than animals having a lower tolerance [49]. The same may hold true for *D. magna* experiencing constant predation stress exerted by *Triops*.

Prohibitin is a ubiquitously expressed and well conserved protein, which is thought to be a negative regulator of cell proliferation in mammalian cells [50]. The similar protein in Drosophila, lethal (2) 37Cc, is most strongly expressed during late embryogenesis and may play a role in cuticle synthesis because of its presence during molts [51]. Therefore it seems possible, that the lower abundance of this protein may reflect changes of cuticle synthesis during the formation of morphological defences in *D. magna*.

The sequence of signal transducer and activator of transcription (STAT) protein is most similar to Stat92E in Drosophila. Stat92E is a signal protein and transcription factor in the well characterised JAK/STAT signalling pathway important for processes such as cellular proliferation, especially during embryonic development, immune response and stem cell maintenance [52]. Interestingly, Stat92E shows opposing influence on cell proliferation depending on developmental stage. During early development, Stat92E promotes cell proliferation whereas in later larval stages it reduces proliferation [53]. The under representation of this protein in late-stage *D. magna* embryos exposed to *Triops* kairomones may reflect a changed cell proliferation pattern during the formation of inducible defences.

### Proteomic evidence for enhanced energy demand and biosynthetic activity as a consequence of kairomone exposure

The more abundant proteins glyceraldehyde-3-phosphate dehydrogenase (GAPDH), fructose-bisphosphate aldolase (Ald), ATP synthase subunit beta (ATPsyn-beta) and nucleoside diphosphate kinase (NDK) are related to energy metabolism and biosynthetic processes (see also Figure 5). Both, GAPDH and Ald are key enzymes of the glycolytic degradation of glucose. In addition, GAPDH provides NADPH for biosynthesis of fatty acids, amino acids and nucleic acids. ATP-Syn-beta is a subunit of ATP-Synthase, which catalyses ATP synthesis within the respiratory chain. NDK provides nucleoside triphosphates for a variety of biosynthetic pathways.

Enhanced biosynthesis has already been reported in *D. magna* exposed to *Chaoborus* or fish kairomones [26]. *D. magna* showed a decreased body length when exposed to the vertebrate predator and an increase in body length when exposed to the invertebrate predator. RNA levels of protein biosynthesis related genes were increased for both treatments with higher levels in the fish kairomone treatment indicating a higher energy demand in predator exposed animals.

Another protein related to energy metabolism is vitellogenin, the precursor of the major yolk protein vitellin. Yolk proteins serve as an energy supply as well as organic building blocks throughout embryonic development of oviparous animals [54]. They are usually synthesised in extra ovarian tissues like the insect fat body [55] or non-mammalian vertebrate liver [56] and are taken up by the developing oocyte. During this process, usually referred to as vitellogenesis, vitellogenin is modified through cleavage, phosphorylation, glycosylation and lipidation [57]. At the time of embryogenesis, yolk proteins are further processed and degraded for embryo nutrition [58].

Due to the various processing steps during vitellogenesis and embryogenesis, the frequent occurrence of different vitellogenin related protein spots in 2D-gels of *D. magna* embryos found in our study is not surprising. Most of the spots were protein fragments with strong isoelectric point (pI) shifts and much smaller molecular weight (MW) compared to theoretical MW (see Additional file 2). Of the 43 vitellogenin-related protein spots found in our proteomic analysis, only 2 proteins were more abundant in *Triops* exposed *D. magna* whereas 41 were less abundant. Therefore, predator exposure seems to influence either the total amount of vitellogenin per egg provided by the mother or the yolk utilisation through the embryo because of higher energy demands.

Other studies also found yolk protein dynamics influenced by predator-released kairomones *D. magna* exposed to fish or *Chaoborus*. The proportion of total yolk used for egg production remained constant [59]. In presence of fish kairomones, *D. magna* reproduced not only earlier and at a smaller body size, but also had a higher number of offspring and this offspring had a smaller body size when compared to a control group [60,61]. In the presence of *Chaoborus*, *D. magna* reached maturity later at an increased body size and had a smaller number of offspring with larger body size [62]. *Triops* kairomones seems to increase both, the number and the size of offspring in *D. magna*[31,63]. Therefore, less yolk may be distributed to a single egg. However, the under representation of vitellogenin spots in kairomone exposed *D. magna* embryos found in this study may also indicate a higher energy demand. In addition, the higher abundance of other proteins related to energy metabolism and biosynthetic processes mentioned previously supports an increased energy demand of the embryo while building up *Triops*-induced defensive structures.

### Kairomone exposure of Daphnia increases levels of proteins necessary for reinforcement of the muscular system

The muscle related proteins actin, troponin and paramyosin were all more abundant in *Triops* exposed *D. magna* embryos (see also Figure 5). Actin was found in four different protein spots with molecular weight (MW) higher than the theoretical value and acidic pI shifts, indicating posttranslational modifications. Additionally, one protein spot had a considerably smaller MW indicating a cleaved fragment (see Table 1). Actin is a major component of the cytoskeleton as well as of muscle fibres and is now one of the most abundant and highly conserved proteins in eukaryotes usually encoded in multiple genes [64].

Comparing the actin sequences using blastp algorithm, the most similar sequence in *D. melanogaster* for daphmag3mtv7094t1 is Act87E (FBgn0000046), whereas daphmag3mtv3l15317t1 was most similar to Act5C (FBgn0000042). Act87E is known to be expressed in the body wall muscles during embryonic, pupal and adult stages while Act5c is a ubiquitous cytoplasmic actin, being expressed throughout all life stages [65]. However, Röper et al. [66] showed that muscle-specific actin is incorporated into cytoplasmic structures, and cytoskeletal actin is incorporated into muscles for all actin paralogues of *D. melanogaster*. Therefore, it is not possible to deduce the function of actin only from its protein sequence.

Actin was connected to the formation of inducible defence in *D. magna* with contradictory results so far. Pijanowska and Kloc [24] reported a strong decrease of actin protein level in *D. magna* exposed to either *Chaoborus* or fish predation using western blot analysis. On the contrary, Schwarzenberger et al. [26] found a moderate increase of actin mRNA expression in *D. magna* exposed to fish and only a slight decrease in *D. magna* exposed to *Chaoborus* using real-time qPCR. These inconsistent results may be a consequence of the different classes of molecules addressed in these studies, since RNA expression is not a reliable surrogate marker for protein expression.

In our proteomic analysis, strong evidence for a higher abundance of one muscle-specific actin and one cytoplasmic actin was found. In addition, two other muscle-specific proteins, troponin and paramyosin were more abundant in *D. magna* exposed to *Triops* kairomones. Troponin is an actin-binding protein found in thin filament of vertebrate and invertebrate muscle where it regulates actomyosin activity in a *Ca*^2+^ dependant manner [67]. Paramyosin is part of the thick filament of invertebrate muscle and a central player in regulating its diameter, with filaments of increased diameter showing an increased paramyosin:myosin ratio [68]. Predator-induced increase of muscle size has been found in other organisms, e.g. in the blue mussel *Mytilus edulis*[69] and in tadpoles of *Rana lessonae*, in the latter case it improved swimming performance. This may also be the case for defended *Daphnia*, as *D. magna* exposed to *Chaoborus* or fish kairomones show increased escape response time and higher behavioural alertness [24]. In addition, increasing muscular mass may also compensate for the consequences of carapace fortification or altered hydrodynamics resulting from a changed carapace morphology.

### Cuticle proteins and chitin-modifying enzymes may cause carapace fortification in kairomone exposed *Daphnia*

In *T. cancriformis* exposed *D. magna* embryos, five proteins related to exoskeleton show a higher abundance. Out of this, three proteins were similar to cuticle proteins and two proteins were similar to chitin-modifying enzymes (see also Figure 5).

The carapace of *D. magna* consists of a chitinous integument folded back on itself with a small haemocoelic space in between. Inner and outer integument are connected by pillars as supporting structures [70]. This integument can be separated in the extracellular cuticle and the cellular epidermis. The cuticle consists of the two layers, epi- and procuticle [71]. In arthropods, epicuticle is mainly built out of proteins and lipids and procuticle is made of chitin filaments embedded in a proteinaceous matrix [72]. The properties of cuticle depend highly on the amount and combination of included proteins [73] and also on the degree of acetylation, which may influence cross-linking between protein matrix and chitin filaments [74].

Searching the sequences of the three cuticle proteins more abundant in kairomone exposed *D. magna* embryos against the prosite database for protein domains ([75], [http://prosite.expasy.org/prosite.html]) revealed chitin-binding domains in all three sequences. Consensus sequences were of the so called R&R type [76], with all proteins containing one or two RR-2 subgroups, usually associated with hard cuticles [77]. In addition, daphmag3mtv3l7285t1 also has a short consensus sequence of the RR-1 type, usually found in soft cuticles.

As further chitin modifying enzymes, we found chitin deacetylase type 1 and 2A in three different spots at around 80 kDa. These two proteins have a very similar molecular weight and pI and were therefore not well discriminated on the 2D-Gel. Molecular weight of these two proteins was 20 kDa higher than expected and pI was slightly smaller than computed pI (see Table 1), which indicates different states of post-translational modifications within the three different spots. Chitin deacetylase is a chitin modifying enzyme, which catalyses N-deacetylation of chitin and therefore changes protein binding affinity of chitin filaments. In *Tribolium castaneum*, several types of chitin deacetylase have been identified, with type 1 and 2 mainly expressed in the exoskeletal epidermis [78]. RNAi experiments revealed lethal phenotypes when using dsRNA corresponding to this chitin deacetylases. Here, animals failed to shed their old cuticles because the new synthesised cuticle lacked mechanical strength [78]. These findings support that these chitin modifying enzymes are involved in forming a harder cuticle in predator exposed *D. magna*.

Fortification of the exoskeleton in response to predator kairomones is known to play a role in inducible defences of some *Daphnia* species. *D. middendorffiana* exposed to the predatory copepod *Heterocope septentrionalis* shows increased cuticle thickness and cuticle strength [19]. Furthermore, *D. pulex* and *D. cucullata* exposed to *Chaoborus* larvae increase cuticle hardness and *D. cucullata* shows increased cuticle thickness and increased diameter of the cuticular pillars [20]. Recently, similar hidden defences were also found in *D. magna* exposed to *Triops* kairomones, revealing increased cuticle hardness, thickness and pillar diameter [21]. Carapace fortification is thought to act as protection against invertebrate predation, e.g. by increasing the escape efficiency of prey when being caught by the predator [20]. Cuticle related proteins with a higher abundance in *D. magna* exposed to *Triops*, i.e. R & R cuticle proteins as well as chitin deacetylases, may be involved in the necessary changes of chitin cross-linking with matrix proteins already in late stage *D. magna* embryos, causing increased carapace stability.

## Conclusion

In our proteomic analysis, we found evidence that proteins related to cuticle, muscular system, energy metabolism and regulatory proteins are involved in the phenotypic plastic changes induced by *Triops* kairomones in *D. magna*. Cuticle proteins and the cuticle modifying enzymes chitin deacetylases 1 and 2A seem to be directly involved in the formation of morphological changes of the carapace, possibly altering chitin cross-linking with matrix proteins and therefore strengthen carapace stability. The same holds true for changes in abundance of muscle proteins (actin, paramyosin and troponin), which may adjust the muscular system to altered carapace morphology and enabling behavioural changes. Furthermore, proteins not directly involved in building up morphological traits were either involved in energy metabolism and biosynthetic processes or had regulatory functions. These proteins may reflect necessary changes in metabolism needed for the formation of inducible defences. The altered levels of regulatory proteins provide first evidence on signalling pathways possibly involved in the formation of inducible defences i.e. the Ras-mediated signalling pathways (14-3-3 *ζ*), glycosylation (Phosphomannomutase), protein folding (Heat shock protein), regulation of cuticle synthesis (Prohibitin) and translation regulation (STAT).

Our holistic proteomic analysis revealed promising candidate proteins involved in phenotypic plastic response of *Daphnia magna* exposed to kairomones of the predator *Triops cancriformis*. Proteins altered in abundance were either directly involved in the formation of defensive traits or reflect involved regulatory or metabolic pathways. Most interestingly, three proteins connected to this inducible defence (cuticle protein, vitellogenin, actin) belong to known tandem duplicated genes in *D. pulex*, a genetical design occurring in elevated numbers in the *D. pulex* and possibly also in the *D. magna* genome [8] which is predicted to play an important role in phenotypic plasticity [22].

Hence, our study fosters the knowledge on the molecular mechanisms of defensive trait formation, i.e. carapace fortification and – even more important – on the costs affiliated with the formation of the defence, since costs are thought to be a crucial premise for the plastic expression of a trait, and therefore a prerequisite for the evolution of phenotypic plasticity.

## Methods

### Induction experiment

All experiments reported in this study were conducted in agreement to the animal protection act of Germany. The induction experiment was carried out using a laboratory cultured clone of *D. magna* (*K*_3_4*J*) originating from a former fish pond near Munich, Germany. This clone shows strong morphological plasticity, i.e. increased body length, increased body width, increased tail spine length and increased carapace strength, in response to *Triops* predation [21,31,32]. A laboratory cultured clonal line of *T. cancriformis* provided by Dr. E. Eder, Zoological Institute, University of Vienna served as the predator. The experimental setup was installed in a climate chamber at a constant temperature of 20°C ± 1°C combined with fluorescent lighting at a constant photoperiod (15 h day : 9 h night).

**Figure 6:**
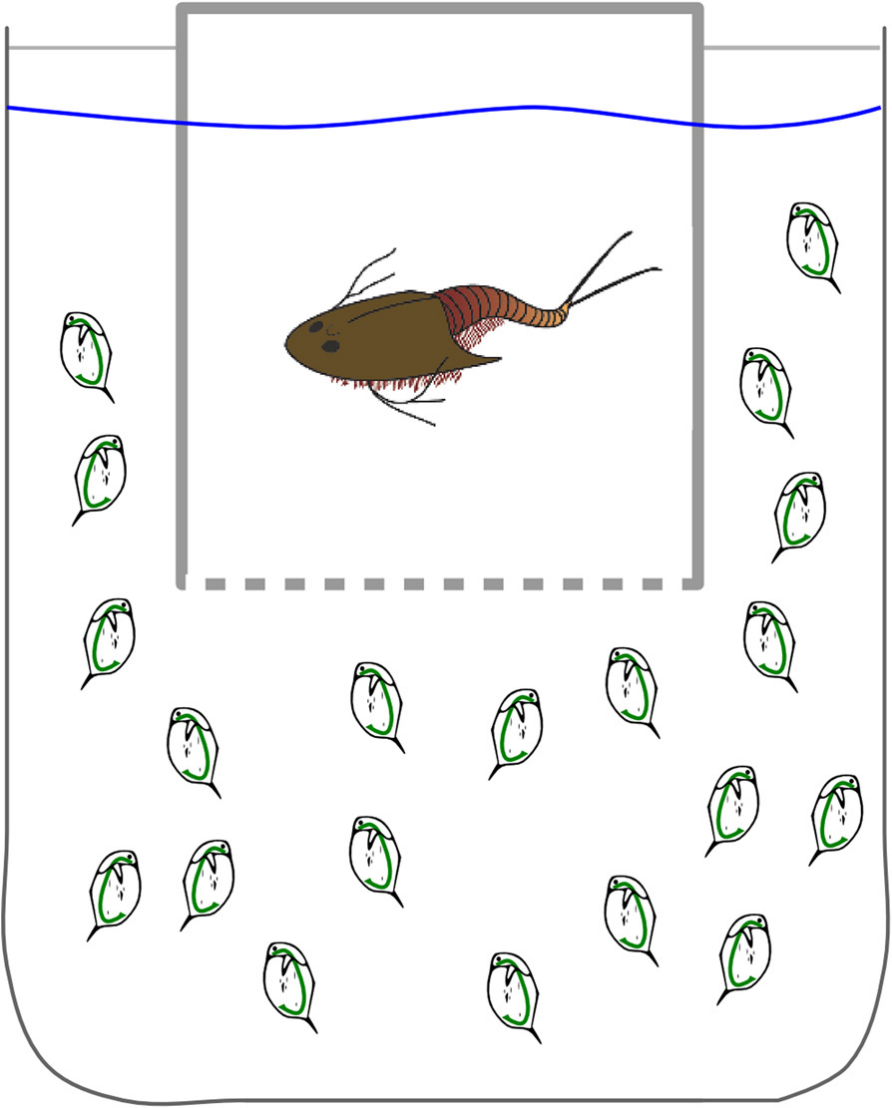
**Setup of induction experiment.** One replicate consists of one beaker with daphnids and a net cage containing the predator, so that daphnids perceive chemical cues of *Triops* but were prevented from being eaten. For the control group, the net cage was empty.

The induction experiment included three biological replicates per group. For each replicate, 20 daphnids were raised in 2 L beakers containing 1.5 L semi-artificial medium [31] and a net cage (mesh width 400 µm; see Figure 6). The net cage contained one *Triops* for the kairomone exposed group allowing chemical cues to pass but preventing the daphnids from getting eaten (one Triops/1.5 L). Dead predators were replaced and feces of the predator were removed on a daily basis. For the control group, a net cage without a predator was placed into the beaker. Every second day, half of the artificial medium was exchanged. Daphnids were fed daily with *Scenedesmus obliquus* at a carbon concentration of 1 mg L^−1^. Triops were also fed every day with living chironomids larvae, and 10 adult dead *D. magna* to take prey-specific alarm cues into account. These cues are released when prey animals are crushed by the predator and are also known to induce defensive structures in *Daphnia*[79]. *Daphnia* were killed using carbon-dioxide saturated water shortly before feeding. Preliminary experiments have shown that chironomids larvae do not induce defences in *Daphnia*.

The timetable of the induction experiment followed previous studies of inducible defences in the *D. magna* -*Triops* system [21,31,32]. The experiment was started by placing 4 age-synchronised randomly chosen primiparous daphnids and one adult *Triops* with a body length between 30 mm and 40 mm into the system. After releasing their first clutch, adult daphnids were removed and neonates were randomly reduced to 20 individuals (F _0_ generation) per beaker. F _0_ mothers were also removed after releasing their first clutch and F _1_ neonates were again reduced to 20 individuals. The same was done after the birth of the next generation (F _2_). The experiment was stopped after three generation cycles (approximately four weeks). After this duration morphological changes are known to be established in all animals of the kairomone exposed group [21,31,32]. In the end, F _2_ generation animals bear their first clutch with embryos of a late developmental stage (black-eye embryos). Hence, age-synchronisation of embryos was in a time range of 12 hours. These embryos were used for proteomic analysis and therefore rinsed out of the mothers‘ brood pouch and washed twice using autoclaved and filtered semi-artificial medium [31] (filter pore size 0.2 µm). Subsequently, embryos were placed into one tube per biological replicate and snap-frozen using liquid nitrogen. Each replicate consisted of 300 – 400 embryos.

### 2D-DIGE

To prepare *Daphnia* embryos for 2D fluorescence difference gel electrophoresis (2D-DIGE) analysis, the frozen samples were homogenised in a mortar under liquid nitrogen thus preventing thawing. The resulting powder was solubilised in lysis buffer (2 mol/L Thiourea, 6 mol/L Urea, 4% CHAPS, 1 cOmplete ULTRA Tablets Mini (Roche) per 5 ml buffer) at a concentration of 1 embryo µL^−1^ buffer. Afterwards, each sample was centrifuged using a QIA Shredder Mini Spin Column (Qiagen) for 2 min at 14,000 g. Sample pH was adjusted to 8.5 using 50 mmol/L NaOH. Protein concentration was analysed by performing a Bradford Protein Assay (Coomassie Plus (Bradford) Assay Reagent, Thermo Scientific) according to the manufacturer’s instructions.

50 µg protein per biological replicate were labelled with 2D-DIGE Cy3 Dye for control or Cy5 Dye for kairomone exposed group (GE Healthcare Life Sciences) following the protocol of the manufacturer. In addition, an internal standard (IPS) was prepared by pooling all biological replicates and labelling 200 µg of this IPS with 2D-DIGE Cy2 Dye.

24 cm gel strips for first dimension isoelectric focusing (IEF) were rehydrated for at least 10 h before starting of IEF with 450 µL rehydration buffer (2 mol/L Thiourea, 6 mol/L Urea, 4% CHAPS, 13 mmol/L DTT, 2% pharmalyte pH 3-10, bromphenol blue).

Prior to IEF, 50 µg of one Cy3-labelled control replicate, 50 µg of one Cy5-labelled kairomone exposed replicate and 50 µg of Cy2-labelled IPS were merged and 65 mmol/L DTT and 2% pharmalyte pH 3-10 were added. This mixed sample was applied via anodic cup loading on one gel strip. IEF was performed using an IPGPhore (Pharmacia Biotech) with a total of 60 kV h per strip.

Before second dimension gel electrophoresis, gel strips were equilibrated for 15 min in 15 mL equilibration buffer (50 mmol/L Tris-HCl pH 6.8, 6 mol/L urea, 30% glycerin, 2% SDS) containing 1% DTT on a shaker (40 min^−1^, Certomat U, Sartorius). Afterwards, a second 15 min equilibration step in 15 mL equilibration buffer with 2.5% iodoacetamide and 200 µL saturated bromphenol blue solution was performed. For second dimension electrophoresis, lab-cast 210 × 260 × 1 mm polyacrylamide gels (1.5 mol/L Tris-HCl pH 8.8, 12.5% acrylamide/bisacrylamide (37.5:1), 0.1% SDS, 0.05% APS, 0.05% TEMED) and an ETTANDaltsix electrophoresis unit (GE Healthcare Life Sciences) were used. Equilibrated gel strips were fixed on top of the gels with the help of 0.5% agarose solved in SDS running buffer (25 mmol/L Tris, 192 mmol/L glycine, 0.2% SDS). Electrophoresis was conducted at 10°C for one hour at 5 W per gel and afterwards at 17 W per gel until the dye front reached the end of the gel.

### Imaging and quantitative analysis

Gels were scanned immediately after electrophoresis using a Typhoon 9400 fluorescence scanner (GE Healthcare Life Sciences) with parameters recommended for 2D-DIGE experiments by the manufacturer. Image analysis and relative quantification were performed with DeCyder™ 2D Software version v7.0 (GE Healthcare Life Sciences). Coordinates of significantly differing protein spots (*p*≤0.05 with FDR correction, *ratio*≥|3| when comparing both treatments) were transferred to a pick list for further processing.

### Excision of spots and tryptic hydrolysis

Gels were stained overnight with Coomassie Brilliant Blue (50% Methanol, 0.5% CBB R-250, 10% acetic acid) and then destained for at least 8 h. Spots of interest were cut out automatically with a PROTEINEER spII robot (Bruker Daltonics) using the created pick list. Next, spots were digested using a DigestPro MS robot (Intavis) with the following protocol: (i) wash step with 60 µL 50 mmol/L *NH*_4_*HCO*_3_, (ii) wash step with 90 µL 50% acetonitrile, 25 mmol/L *NH*_4_*HCO*_3_, (iii) 20 min wash in 60 µL acetonitrile, (iv) 20 min wash in 60 µL 50 mmol/L *NH*_4_*HCO*_3_, (v) 20 min wash in 60 µL acetonitrile, (vi) 15 min wash in 60 µL acetonitrile, (vii) addition of 90 ng porcine trypsin (Promega) in 15 µL 50 mmol/L *NH*_4_*HCO*_3_ and incubation at 37°C for 6 h, (viii) addition of 15 µL 2.5% formic acid. Samples were than dried in a vacuum centrifuge (Vacuum Concentrator, Bachofer) and stored at -20°C until mass spectrometric analysis.

### LC-MS/MS analysis

Nano-flow liquid chromatography tandem mass-spectrometry (nano-LC MS/MS) was performed with a nano LC ultra chromatographic device (Eksigent) coupled to a LTQ mass spectrometer (Thermo Scientific). Samples were resolved in 0.1% formic acid under 10 min sonication (Sonorex RK100, Bandelin). Subsequently, 10 µL of each sample were injected and loaded on a C18 trap column (C18 PepMap100, particle size: 5 µm, 100 Å, column size: 300 µm × 50 mm, Dionex) for 10 min at a flow rate of 5µmin^−1^ using mobile phase A (0.1% formic acid). RP chromatography was done at a flow-rate of 280nLmin^−1^ using a Reprosil-Pur C18 separation column (Reprosil-Pur C18 AQ, 3 µm, 150 mm × 75 µm (ID), Dr. Maisch) with a 30 min linear gradient from 0% to 60% mobile phase B (A: 0.1% formic acid, B: 84% acetonitrile and 0.1% formic acid). For electrospray ionisation a distal coated Silica Tip (FS-360-50-15-D-20, New Objective) with a needle voltage of 1.4 kV was used. The MS method consisted of a cycle combining one full MS scan (Mass range: 300 – 2000 m/z) with three data dependant MS/MS events (35% collision energy). The dynamic exclusion was set to 30 s.

### Bioinformatic processing

The MS/MS data were searched with Mascot Version: 2.3.00 (Matrix Science) using the following parameters: i) Enzyme: Trypsin; ii) Fixed Modification: Carbamidomethyl (C); iii) Variable modifications: Oxidation (M); iv) Peptide tol. 2 Da; v) MS/MS tol. 0.8 Da; vi) Peptide charge 1+, 2+ and 3+; vii) Instrument ESI-TRAP and viii) Allow up to 1 missed cleavages. As database, pre-released gene-predictions of *D. magna* (V2.4 effective 05/2012) were used. These sequence data were produced by The Center for Genomics and Bioinformatics at Indiana University and distributed via wFleaBase in collaboration with the Daphnia Genomics Consortium (http://daphnia.cgb.indiana.edu). Here, redundant entries of 90% similarity or more were detected through the software cd-hit [80] and removed. In addition, a common contaminants database (Max Planck Institute of Biochemistry, Martinsried, Germany: http://maxquant.org/contaminants.zip) was added. Mascot data were further processed with Scaffold 3 (Proteome Software), here “Protein Probability” and “Peptide Probability” were set to 99% and at least 2 unique peptides were used for protein identification.

Data were further processed with customised R scripts [81] (see also Additional file 5). Protein sequences were compared to data of NCBI nr [82] database using the NCBI Basic Local Alignment Search Tool (BLAST, *e*−*value*<0.001) algorithm with R Package Bio3d [83].

GI numbers resulting from NCBI nr search were converted to UniProt accession numbers and further processed using the R biomaRt package [84] to gain further information on protein names and annotations, which are not yet available for preliminary *D. magna* sequence data. If no meaningful protein name was available for the first blast hit, which means that the protein name was either “uncharacterised” or a alphanumeric combination, further results were searched and added to the protein result. In addition, FlyBase Gene ID was looked up for the first blast hit related to *Drosophila melanogaster*.

Hierarchical clustering and heatmap were generated using the R package gplots. Cluster analysis of protein annotation (two-sided hypergeometric with Benjamini-Hochberg correction) and network visualisation (kappa-score ≥0.3) were performed using the software Cytoscape 2.8.3 [85] with the ClueGO plug-in v1.7 [39] using the Gene Ontology and KEGG databases for *D. melanogaster* and CluePedia plug-in v1.0.8 [86].

Protein data were compared to known tandem duplicated genes in *D. pulex*[8], summarised in http://wfleabase.org/genome-summaries/gene-duplicates/ daphnia_tandemgene_table.html.

## Competing interests

The authors declare that they have no competing interests.

## Authors’ contributions

CL, TF and GJA designed the study. CL conducted the induction experiment and provided samples for proteomic analysis. KAO conducted proteomic experiments, performed mass spectrometry analysis and conducted bioinformatic analysis of the data. TF supervised mass spectrometry analysis. KAO wrote the first draft of the manuscript and CL, TF and GJA contributed substantially to revisions. All authors read and approved the final manuscript.

## Authors’ information

Georg J Arnold and Christian Laforsch share senior authorship.

## Supplementary Material

Additional file 1 **Spectral counting data.** Spectral counting data, resulting from analysis of mass-spectrometric raw files with Scaffold Software, for all analysed spots as compressed zip file, for more details see Additional file 3. Click here for file

Additional file 2 **Spot data.** Data of all identified spots, for more details see Additional file 3. Click here for file

Additional file 3 **Readme.** Readme explaining contents of supporting files in more detail. Click here for file

Additional file 4 Overlay images of 2D-DIGE-Gels. Click here for file

Additional file 5 R-scripts. Click here for file
