# Supplementary material for: Proteomic analysis of Daphnia magna hints at molecular pathways involved in defensive plastic responses
Source: BMC Genomics. 2014 Apr 24;15:306. doi: 10.1186/1471-2164-15-306 (PMC4236883; doi:10.1186/1471-2164-15-306)
Supplement: Additional file 2 — Spot data. Data of all identified spots, for more details see Additional file 3. [file 1471-2164-15-306-S2.pdf › spotdata/pick2/readme.docx]

#######Kathrin Otte 11/2013########

.tab/xls files refer to spectral counting data generated with Scaffold for all analysed spots (first run) with columns:

1 database description (in case of D. magna database usually not that informative); 2 Accession number of D. magna database; 3 Did Scaffold detect an isoform?; 4 Molecular weight of Protein in kDa; each of the following columns represent spectral counting data of analysed spot, header contains pick spot number.

KO89709-decyder.txt first column contains unique Dige spot numbers, rows indicate pick spot number used in .tab files.

ratiosd.txt contains 1 unique Dige spot numbers, 2 computed mean of spot intensity, 3 computed standard deviation of spot intensity, 4 corrected p value of decyder Ttest, 5 mean computed by decyder for control purpose
