# Supplementary material for: Proteomic analysis of Daphnia magna hints at molecular pathways involved in defensive plastic responses
Source: BMC Genomics. 2014 Apr 24;15:306. doi: 10.1186/1471-2164-15-306 (PMC4236883; doi:10.1186/1471-2164-15-306)
Supplement: Additional file 3 — Readme. Readme explaining contents of supporting files in more detail. [file 1471-2164-15-306-S3.zip]

##########Kathrin Otte 11/2013############

T1 - spotdata of all identified spots with columns 1 pick spot number (myspot); 2 unique dige spot number used in the article; 3 Uniprot ID of first blast hit; 4 Protein Name and organism of first blast hit and if protein name not informative, next blast hit with convincing protein name and corresponding organism; 5 SwissprotID of first Swissprot Hit, 6 ID of first flybase hit for Drosphila melanogaster; 7 mean Ratio of spot intensity compared to internal pooled standard; 8 standrad deviation of spot intensity compared to internal pooled standard; 9 spectral count for protein in MS/MS analysis; 10 How many IDs per spot?; 11 theoretical molecular weight; 12 molecular weight on 2D-Gel; 13 theoretical pI; 14 pI on 2D-Gel

F1 - coloured overlay images of all three 2D-DIGE-Gels used in this study (colours are not corrected for intensity of internal pooled standard)

S1 - compressed Scaffold spectral counting spotdata for all picked spots, two runs of spot picking were conducted, each directory refers to one pick run, detailed information can be found in extra readme.txts located in each directory

S2 - this file
