# Supplementary material for: Proteomic analysis of Daphnia magna hints at molecular pathways involved in defensive plastic responses
Source: BMC Genomics. 2014 Apr 24;15:306. doi: 10.1186/1471-2164-15-306 (PMC4236883; doi:10.1186/1471-2164-15-306)
Supplement: Additional file 4 — Overlay images of 2D-DIGE-Gels. [file 1471-2164-15-306-S4.docx]

Supplementary Material Otte et al. 2013: F1

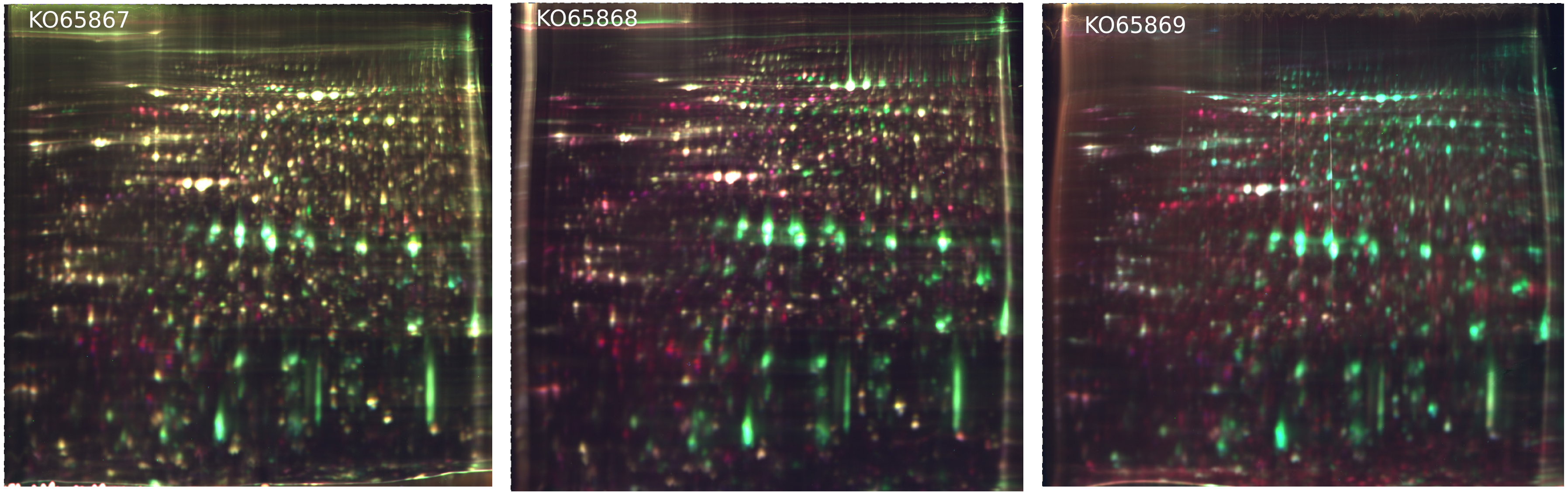

Figure 1: F1: 2D-DIGE gels overlay images of three biological replicates. Control group was labeled with Cy3 (green), *T. cancriformis* exposed group was labelled with Cy5 (red), internal pooled standard was labelled with Cy2 (blue).
